# Supplementary material for: Higher convergence of human-great ape enteric eukaryotic viromes in central African forest than in a European zoo: a One Health analysis
Source: Nat Commun. 2023 Jun 21;14:3674. doi: 10.1038/s41467-023-39455-9 (PMC10282056; doi:10.1038/s41467-023-39455-9)
Supplement: Supplementary file 1 — Supplementary Information [file 41467_2023_39455_MOESM1_ESM.pdf]

Supplementary Information

Higher convergence of human-great ape enteric viromes in central African forest than in a European zoo: A One Health analysis

This supplementary file contains 3 supplementary tables, 2 supplementary figures, 2 supplementary method, and 1 supplementary result.

Supplementary Table 1: Pairwise comparisons between groups using permutation MANOVAs on a Bray Curtis distance matrix (a) and a weighted UniFrac distance matrix (b). Each group is defined by its habitat site (Cam = Cameroon, Zoo = European Zoo) and animal species (Chimp = Chimpanzee, Gor = Gorilla, Hum = Human).

a)

	**CamChim**	**CamGor**	**CamHum**	**ZooChim**	**ZooGor**
**CamGor**	0.0017	-	-	-	-
**CamHum**	0.0017	0.0017	-	-	-
**ZooChim**	0.0041	0.0017	0.0017	-	-
**ZooGor**	0.0017	0.0017	0.0017	0.0161	-
**ZooHum**	0.0046	0.0017	0.0190	0.0046	0.0030

b)

	**CamChim**	**CamGor**	**CamHum**	**ZooChim**	**ZooGor**
**CamGor**	0.0017	-	-	-	-
**CamHum**	0.0025	0.0017	-	-	-
**ZooChim**	0.0032	0.0017	0.0017	-	-
**ZooGor**	0.0025	0.0017	0.0017	0.0060	-
**ZooHum**	0.0025	0.0017	0.0017	0.0032	0.0017

Supplementary Table 2: Viral reads information among the different groups

	Min.	1st Q	Median	Mean	3rd Q	Max.
CamChim	1077	1228	2027	17795	3074	97252
CamGor	42.56	103.40	358.80	495.59	636.78	2192.54
CamHum	38.33	70.01	232.17	2052.74	578.30	19386.06
ZooHum	95.58	150.85	265.97	8024.99	5812.55	43886.62
ZooChim	632.2	677.9	764.4	1226.1	1698.1	2357.7
ZooGor	3134	3648	16152	19561	30109	47685

All reads were expressed in RPM (read per million of sequenced reads))

Supplementary Table 3: Accession numbers of viral sequences used for the network analysis in Figure 4

**Name**	**Accession Number**	**Name**	**Accession Number**
HAdV-A12	NC001460.1	SAdV-C31	FJ025904.1
HAdV-A18	GU191019.1	SAdV-E22	AY530876.1
HAdV-A31	AM749299.1	SAdV-E23	AY530877.1
HAdV-B11	NC011202.1	SAdV-E24	AY530878.1
HAdV-B14	AY803294.1	SAdV-E25	AC000011.1
HAdV-B16	AY601636.1	SAdV-G1	NC006879.1
HAdV-B21	AY601633.1	SAdV-G7	DQ792570.1
HAdV-B3	NC011203.1	Enterovirus A	AY421760.1
HAdV-B34	AY737797.1	Enterovirus B	NC_038307.1
HAdV-B35	AY271307.1	Enterovirus C	V01149.1
HAdV-B7	AC000018.1	Enterovirus D	AY426531.1
HAdV-C1	AF534906.1	Enterovirus E	D00214.1
HAdV-C2	NC001405.1	Enterovirus F	DQ092770.1
HAdV-C5	AC000008.1	Enterovirus G	AF363453.1
HAdV-C6	HC492785.1	Enterovirus H	AF326759.2
HAdV-D36	GQ384080.1	Enterovirus J	AF326766.2
HAdV-D37	DQ900900.1	Enterovirus K	KX156158.1
HAdV-D46	AY875648.1	Enterovirus L	KU587555.1
HAdV-D48	EF153473.1	Rhinovirus A	FJ445111.1
HAdV-D49	DQ393829.1	Rhinovirus B	DQ473485.1
HAdV-D53	FJ169625.1	Rhinovirus C	EF077279.1
HAdV-D54	NC012959.1		
HAdV-D8	AB448767.1		
HAdV-D9	NC010956.1		
HAdV-E4	NC003266.2		
HAdV-F40	NC001454.1		
HAdV-F41	DQ315364.2		
HAdV-G52	DQ923122.2		
SAdV-A1	NC006144.1		
SAdV-A48	HQ241818.1		
SAdV-A6	CQ982401.1		
SAdV-B21	AC000010.1		

Supplementary Figure 1: Distribution of viral reads according to virus host. Each group is defined by its habitation site (Cam=Cameroon, Zoo=European Zoo) and animal species (Chimp=Chimpanzee, Gor=Gorilla, Hum=Human). Source data are provided as a Source Data file.

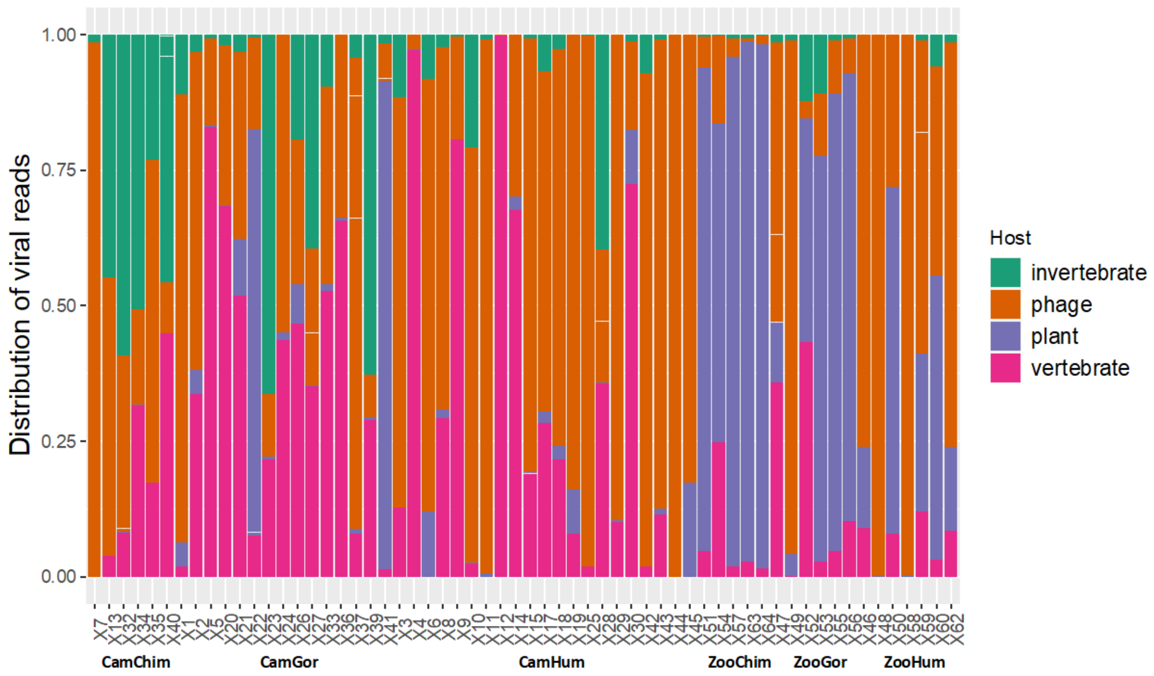

Supplementary Figure 2: Boxplot of diversity indices for (a) all viruses and (b) vertebrate viruses. Within each box, the center line denotes the median value (50th percentile) whereas the box extends from the 25th to the 75th percentile; whiskers mark the maximum and minimum values. N-values are shown above the x-axis. Two-sided Wilcoxon test significance (p<0.05) between each group is presented. Source data are provided as a Source Data file.

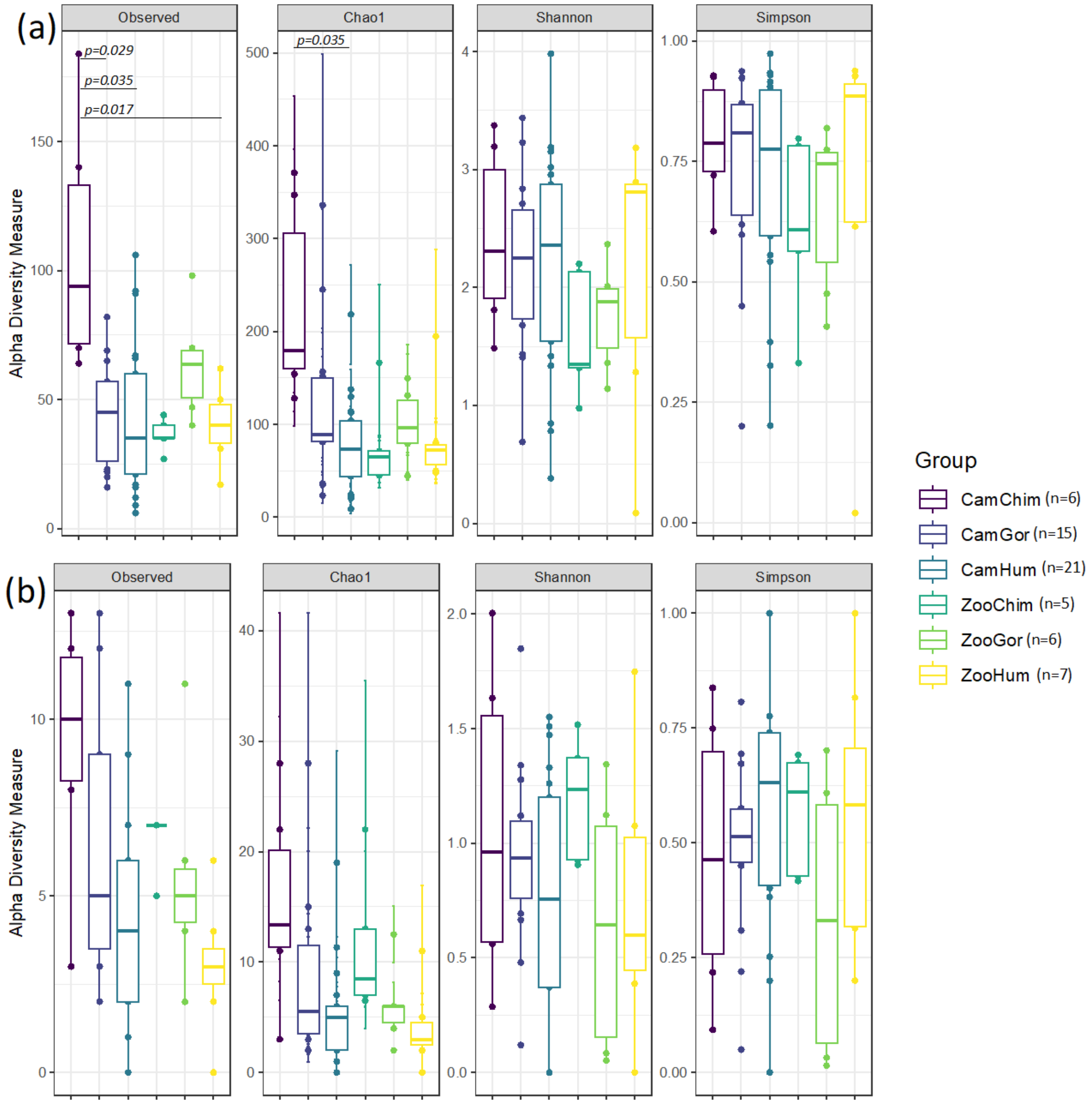

Supplementary Methods 1: Virome bioinformatics analysis based on NCBI nt database

Raw reads were cleaned using TRIMMOMATIC. Duplicated reads were removed using Dedupe. After alignment with BWA on human, chimpanzee or gorilla genomes according to animal species, taxonomic assignment of unaligned reads was carried out using Kraken2 with NCBI nt database.

Alpha and Beta Diversity analysis were conducted using packages Phyloseq v1.22.3 and Vegan v2.5-4 in R v3.4.4[68,69]. For Alpha diversity, Simpson, Shannon, Chao1 and Richness index were calculated. For Beta diversity, Bray Curtis dissimilarity and Unifrac metrics were used. Principal coordinate analysis (PCoA) and network analysis was done with either Bray Curtis or weighted Unifrac distance. Permutational analysis of variance (PERMANOVA) was used to compare microbial communities between each group based on Bray Curtis dissimilarity indices, weighted and unweighted Unifrac distances using the adonis2 function with R package vegan.

Viral taxa shared between the human group and at least one great ape group (chimpanzee or gorilla) in Cameroon and in the zoo were identified.

Supplementary Results 1: Virome bioinformatics analysis based on NCBI nt database

Fecal sample characterization and comparison

We focused on vertebrate viruses because of their importance for viral transmission and emergence. Among vertebrate viruses, 40 families, 106 genus and 442 species were identified. The number of samples positive for each virus family among the ten most prevalent viral families is detailed in Table 1. All the ten most abundant families were identified in all human, gorilla and chimpanzee groups (highlighted in grey in Table 1).

Supplementary Table 4: Number (%) of samples found positive for one of the vertebrate viral families by group. Each group is defined by its habitation site (Cam=Cameroon, Zoo=European Zoo) and animal species (Chimp=Chimpanzee, Gor=Gorilla, Hum=Human).

	CamChim (n=5)	CamGor (n=15)	CamHum (n=22)	ZooChim (n=6)	ZooGor (n=6)	ZooHum (n=7)
Parvoviridae	6	15	22	6	6	7
Picobirnaviridae	6	15	22	5	6	7
Herpesviridae	6	11	19	6	6	6
Paramyxoviridae	5	11	22	5	6	5
Picornaviridae	5	12	19	1	6	6
Matonaviridae	6	11	20	1	3	6
Adenoviridae	4	11	15	6	4	2
Retroviridae	5	11	12	3	3	4
Caliciviridae	1	5	15	4	6	4
Reoviridae	2	7	17	3	3	3
Arenaviridae	4	9	10	2		4
Genomoviridae	4	6	9	2	3	2
Flaviviridae	1	8	11		3	1
Peribunyaviridae		8	12		1	2
Arteriviridae		2	9	2	6	3
Coronaviridae	2	5	4	5	3	
Poxviridae	1	6	7		1	4
Papillomaviridae	3	4	7		1	2
Hepeviridae		6	8		1	
Astroviridae	1	3	7		1	1
Circoviridae	4		5	2		2
Phenuiviridae	2	1	7		1	2
Hantaviridae		2	5			
Orthomyxoviridae			6	1		
Pneumoviridae	1	3	2		1	
Iflaviridae	6					
Nairoviridae	1	1	4			
Nyamiviridae	6					
Hepadnaviridae			2	2	1	
Marnaviridae		3	1		1	
Rhabdoviridae		1	2			1
Tobaniviridae			4			
Alloherpesviridae		3				
Asfarviridae		1	1		1	
Nodaviridae	2				1	
Polyomaviridae			3			
Togaviridae		1			1	
Bornaviridae						1
Filoviridae		1				

Viral diversity and composition

Global virus richness was significantly higher (p<0.01) in Cameroon gorilla than among Cameroonian humans and Zoo apes (Shanon and Simpson index), although Chao and Observed indeses were significantly higher in Zoo gorilla than Cameroon gorilla (Figure 1A). When we focused only on vertebrate viruses, no significant difference was observed between the six different groups, despite a tendency for higher diversity in Cameroon chimpanzees (Figure 1B).

Supplementary Figure 3: Boxplot of diversity indices for all virus (a) and for vertebrate viruses (b). Within each box, the center line denotes the median value (50th percentile) while the box extends from the 25th to the 75th percentile; whiskers mark the maximum and minimum values. N-values are shown above the x-axis. two-sided Wilcoxon test significance between each group is presented. Source data are provided as a Source Data file.

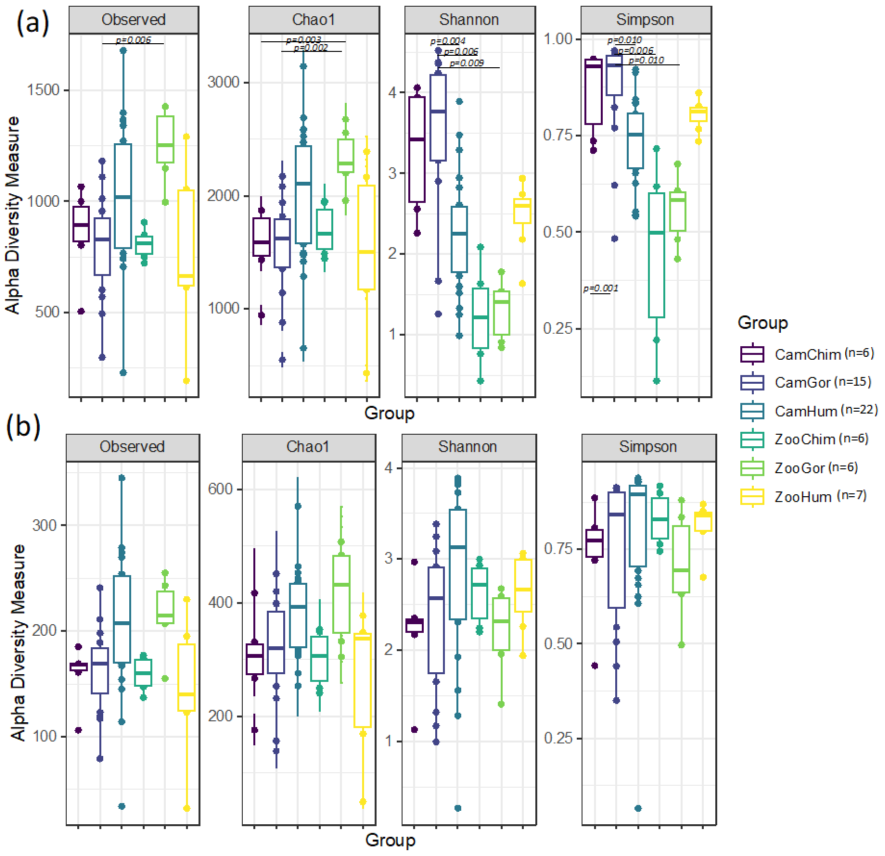

The comparisons of virome composition using a PERMANOVA with Bray Curtis dissimilarity indices and weighted unifrac showed that the virome differed significantly across different groups (p values < 0.001). An unsupervised analysis with a PCA among all samples showed that the global virome

composition differed between host species, with a closer similarity between zoo chimpanzees and gorillas (Figure 2a and 2b). The network projection of virome similarity confirmed the proximity between viromes of zoo great apes. Viromes of zoo great apes resembled that of Cameroonian chimpanzees, which in turn was close to that of Cameroonian gorillas. Despite the distinct environments in which stools were collected, the Cameroonian and zoo human viromes closely resembled one another. The network analyses revealed resemblance between Cameroonian humans and Cameroonian gorillas (Figure 2c).

Supplementary Figure 4: PcoA analysis based on Bray-Curtis distances (a) and on weighted Unifrac distances (b). (c) Network plot based on Bray-Curtis distances showing similarities among all sample virome profiles. Only edges connecting individuals (i.e., nodes) with > 10% similarity in their virome are shown. Each group is defined by its habitation site (Cam=Cameroon, Zoo=European Zoo) and animal species (Chimp=Chimpanzee, Gor=Gorilla, Hum=Human). Source data are provided as a Source Data file.

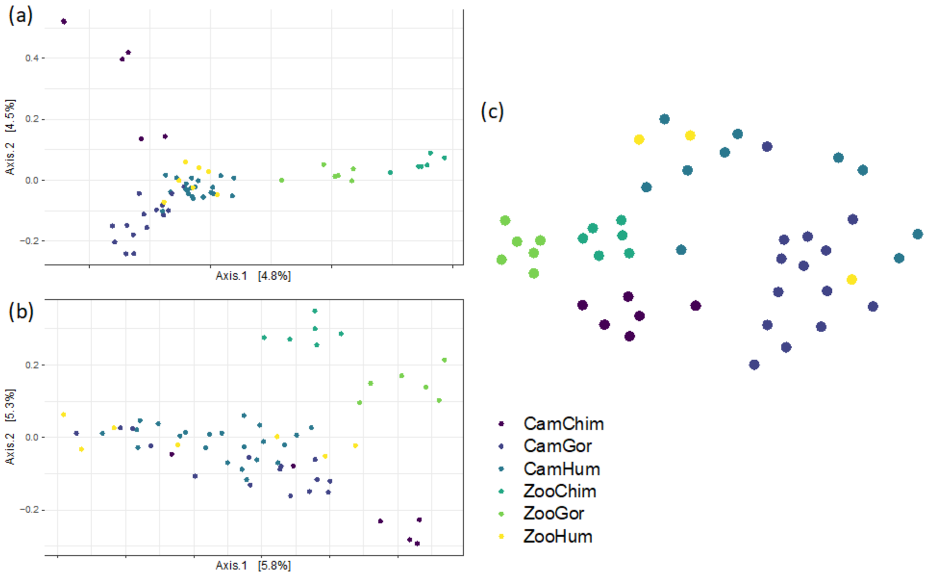

Viral sharing

We investigated whether within a shared environment, great apes and humans might harbor viruses with same lowest-common-ancestor (LCA) taxa identified. In Cameroon, a total of 95 vertebrate viral LCA taxa identified in human stool samples were also found in those of chimpanzees or gorillas: 91 viral LCA taxa in gorilla and human stools, 45 in chimpanzee and human stools, and 41 shared by all 3 groups (Figure 3a). In the European zoo, 41 viral LCA taxa were shared between humans and apes. Viral LCA taxa of each intersection are detailed in supp table.

Supplementary Figure 5: **Venn Diagrams of vertebrates viral lowest-common-ancestor (LCA) taxa identified in human and great ape stools in Cameroon (a) and European zoo (b).** Each group is defined by its habitation site (Cam=Cameroon, Zoo=European Zoo) and animal species (Chimp=Chimpanzee, Gor=Gorilla, Hum=Human). Source data are provided as a Source Data file.

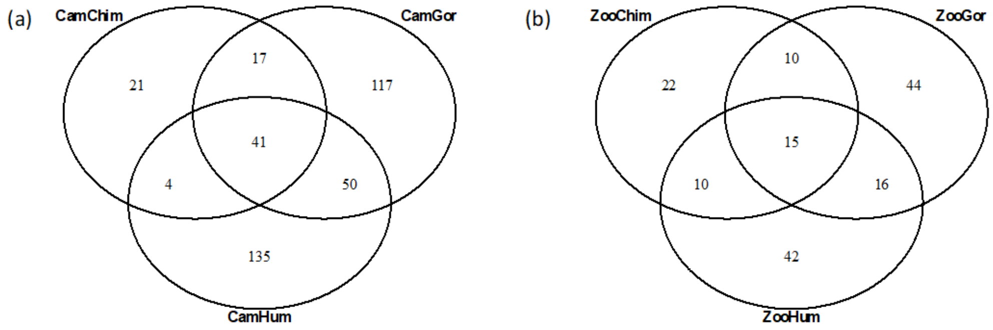

For the phylogenetic analysis, all reads assigned as Adenoviridae were assembled into contigs with Spade. Each generated contig generated were annotated using Geneious Prime (https://www.geneious.com/prime/). Contigs corresponding to the hexon coding gene (6 individuals) were selected for further analysis. They were aligned with simian and human adenovirus reference sequences (Supplementary Table 4) with MAFFT (https://mafft.cbrc.jp/alignment/software/). Phylogenetic analysis was performed by using Iqtree v. 2.1.3 (http://www.iqtree.org/) using 1000 bootstrap replicates. Tree visualization was performed with iTol (https://itol.embl.de/).

Phylogenetic analysis (Figure 3) confirmed the human or simian adenovirus species as defined with the network (Species B for individual 47 (ZooGor), species D for individuals 12, 30 (Cameroon humans) and 36 (Cameroon Gorilla) and species E for individuals 54 and 51 (ZooCHim)). Finally, the tree showed that adenovirus strains from individuals 12 (Cameroon human) and 36 (Cameroon Gorilla) were phylogenetically close with a bootstrap value of 100%.

Supplementary Figure 6: Phylogenetic Tree of adenovirus hexon coding sequences. This phylogenetic tree was constructed using iqtree2 with 1000 bootstraps. The grey circle indicates branches with a support bootstrap value higher than 99%. The color of the sequence labels of this study depends on the group. Each group is defined by its habitation site (Cam=Cameroon, Zoo=European Zoo) and species (Chimp=Chimpanzee, Gor=Gorilla, Hum=Human)

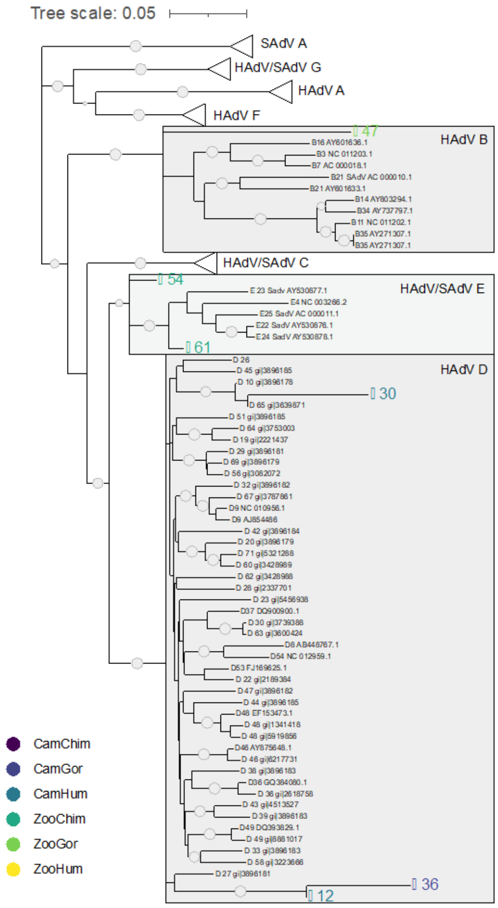

Supplementary Methods 2: Semi-directed interview guides

For great ape zookeepers

1. How long have you worked at this zoo?
2. How long and in what capacity have you worked with this species of great ape?
3. Can you describe for me a typical day working with these animals?
4. Do your daily activities change seasonally, and if so, how?
5. Can you tell me more about feeding of these animals? (timing, dietary composition and quantities for each animal by age and sex, seasonal changes, other relevant practices)
6. From where do you obtain these foods?
7. How do you organize their time outdoors? Are there ever times when they do not go outdoors?
8. Do they eat plant material in the outdoor enclosure or drink from the canal around the island?
9. Do you provide additional supplements from time to time? What?
10. Can you describe any daily physical interactions that you have with these animals? Do you ever touch them? When and why?
11. Have these great apes been sick recently (within the last two months)? If so, which one(s)? What did you do? Did you provide treatment, and if so, what?
12. In your experience, is it possible for disease to pass between humans and captive animals in this zoo setting? Why or why not? Have you ever witnessed such transmission, and if so, when?

For inhabitants of southeastern Cameroon

1. What environmental changes have you observed or heard about in this forest? Over what period of time? What do you think of these changes?
2. In your language, how do you refer to a gorilla? A chimpanzee? Are there terms for referring to both gorillas and chimpanzees? Or terms to denote gorillas, chimpanzees, and monkeys?
3. Where do gorillas/chimpanzees reside in this forest? What are their preferred habitats? Do they remain in the same location, or do they move?
4. Do you find gorillas/chimpanzees in close proximity to your village (use map)?

5. Do you hunt gorillas or chimpanzees? Were these animals hunted in the past? When, and by whom?
6. Who hunts gorillas/chimpanzees now?
7. What weapons are used to hunt gorillas/chimpanzees?
8. How are gorillas or chimpanzees usually butchered? Where and by whom?
9. What do you do with the different parts of the animals?
10. Do people currently keep chimpanzees or gorillas as pets? Of what age? Why or why not? Has this practice of petkeeping changed over time?
11. Can you describe for me the qualities/personality traits of gorillas/chimpanzees?
12. How do they behave with one another? Have you observed their behavior? What features strike you as most important?
13. Do gorillas/chimpanzees have certain capacities or powers? Can you describe them? Have these capacities or powers changed over time?
14. Do gorillas/chimpanzees have knowledge? Of what?
15. What do people think about these animals?
16. How do you behave when you meet one? Why?
17. How do gorillas/chimpanzees behave when they see you?
18. Do people and gorillas/chimpanzees share certain capacities or knowledge?
19. Do you think that relations between people and gorillas/chimpanzees have changed? If so, since when? And why?
20. Do gorillas/chimpanzees ever fall ill? How do you know that they are ill? What are their symptoms?
21. What do gorillas/chimpanzees do when they are ill? How do they care for themselves? (If animal uses leaves for self-treatment, ask person to show leaves. Ask if people use these leaves as well.
22. Is it possible for an illness to be transmitted from a gorilla/chimpanzee to a human? Or from a human to a gorilla/chimpanzee? If so, which illnesses? How does this transmission take place?
