# Supplementary material for: Higher convergence of human-great ape enteric eukaryotic viromes in central African forest than in a European zoo: a One Health analysis
Source: Nat Commun. 2023 Jun 21;14:3674. doi: 10.1038/s41467-023-39455-9 (PMC10282056; doi:10.1038/s41467-023-39455-9)
Supplement: Supplementary file 3 — Description of Additional Supplementary Files [file 41467_2023_39455_MOESM3_ESM.docx]

**Description of Additional Supplementary Files**

**Higher convergence of human-great ape enteric viromes in central African forest than in a European zoo: A One Health analysis**

File name: Supplementary_Data_1

Description: Metadata of stool samples and sequencing information for DNA and RNA sequencing. Each group is defined by its habitat site (Cam = Cameroon, Zoo = European Zoo) and animal species (Chimp = Chimpanzee, Gor = Gorilla, Hum = Human).

File name: Supplementary_Data_2

Description: Main metrics of Adenoviridae and Picornaviridae contigs used in the sequence similarity network representation of Figure 5. For each contig, the length, number of raw reads, and Blastn Best hit are provided.
